# Understanding Design Approaches and Evaluation Methods in mHealth Apps Targeting Substance Use: Protocol for a Systematic Review

**DOI:** 10.2196/35749

**Published:** 2022-07-28

**Authors:** Sahiti Kunchay, Ashley N Linden-Carmichael, Stephanie T Lanza, Saeed Abdullah

**Affiliations:** 1 College of Information Sciences and Technology The Pennsylvania State University University Park, PA United States; 2 Edna Bennett Pierce Prevention Research Center The Pennsylvania State University University Park, PA United States

**Keywords:** substance use, mHealth, human-centered design, use disorder, substance, design rationale, theoretical framing, protocol, systematic review, mobile health, smartphone, mobile phone, digital health

## Abstract

**Background:**

Substance use and use disorders in the United States have had significant and devastating impacts on individuals and communities. This escalating substance use crisis calls for urgent and innovative solutions to effectively detect and provide interventions for individuals in times of need. Recent mobile health (mHealth)–based approaches offer promising new opportunities to address these issues through ubiquitous devices. However, the design rationales, theoretical frameworks, and mechanisms through which users’ perspectives and experiences guide the design and deployment of such systems have not been analyzed in any prior systematic reviews.

**Objective:**

In this paper, we systematically review these approaches and apps for their feasibility, efficacy, and usability. Further, we evaluate whether human-centered research principles and techniques guide the design and development of these systems and examine how the current state-of-the-art systems apply to real-world contexts. In an effort to gauge the applicability of these systems, we also investigate whether these approaches consider the effects of stigma and privacy concerns related to collecting data on substance use. Lastly, we examine persistent challenges in the design and large-scale adoption of substance use intervention apps and draw inspiration from other domains of mHealth to suggest actionable reforms for the design and deployment of these apps.

**Methods:**

Four databases (PubMed, IEEE Xplore, JMIR, and ACM Digital Library) were searched over a 5-year period (2016-2021) for articles evaluating mHealth approaches for substance use (alcohol use, marijuana use, opioid use, tobacco use, and substance co-use). Articles that will be included describe an mHealth detection or intervention targeting substance use, provide outcomes data, and include a discussion of design techniques and user perspectives. Independent evaluation will be conducted by one author, followed by secondary reviewer(s) who will check and validate themes and data.

**Results:**

This is a protocol for a systematic review; therefore, results are not yet available. We are currently in the process of selecting the studies for inclusion in the final analysis.

**Conclusions:**

To the best of our knowledge, this is the first systematic review to assess real-world applicability, scalability, and use of human-centered design and evaluation techniques in mHealth approaches targeting substance use. This study is expected to identify gaps and opportunities in current approaches used to develop and assess mHealth technologies for substance use detection and intervention. Further, this review also aims to highlight various design processes and components that result in engaging, usable, and effective systems for substance use, informing and motivating the future development of such systems.

**International Registered Report Identifier (IRRID):**

DERR1-10.2196/35749

## Introduction

More than 275 million people worldwide used substances in 2021, with problematic substance use costing the lives of half a million people globally in 2019 [1], an increase of more than 20% in the past 10 years. Further, substance use often has far-reaching consequences on human health and well-being, resulting in the loss of 18 million years of healthy life [1]. However, there remains a huge treatment gap—people with substance use concerns, especially in underserved populations (eg, ethnic and racial minorities, individuals experiencing homelessness, and sexual and gender minorities), often do not have access to appropriate diagnoses and care. As such, there has been an increasing focus on addressing this treatment gap by using technology to make substance use detection and intervention delivery more affordable and accessible to individuals and communities. Even with recent advances [2,3], a considerable amount of work remains to ensure that these technological approaches are scalable and usable for all, especially in underserved populations [4].

Earlier efforts to summarize the current research in this domain have primarily focused on efficacy and usability [2,3,5], but few have investigated the design and evaluation approaches that inform these systems and apps. The design principles these detection and intervention apps follow, the theoretical constructs that underlie these systems, and the mechanisms through which users’ perspectives and experiences guide the design and deployment of such systems are individually reported in various works but have not been analyzed in any prior systematic reviews. Understanding these fundamental aspects of mobile health (mHealth) apps for substance use could guide researchers, designers, developers, and even policy makers and provide actionable insights for them in creating effective and usable technological systems for problematic substance use detection and interventions.

Further, an investigation of scalability and ethics, as well as stigma and privacy concerns of various stakeholders of these apps, could have broad implications not only for the domain of mHealth for substance use but also for the broader digital health domain. A few systematic reviews that focus on aspects of users’ experiences with mHealth apps have generated useful findings such as recommendations about improving overall usability [6], capturing engagement in various settings [7,8], and ensuring the privacy and security of users’ data [9,10]; however, all such evaluations have tended to focus on singular aspects of users’ experiences (eg, only evaluating privacy, or only evaluating engagement or usability). So far, no systematic review offers a comprehensive evaluation of how these myriads of designs and considerations are associated with one another, and more importantly, with the intended health outcomes.

Toward the goal of identifying, analyzing, and summarizing these existing systems, we aim to systematically review approaches in the substance use domain of mHealth technologies for the following features: their design techniques; evaluation methodologies; resulting feasibility, efficacy, usability, and overall user experience; exploration of stigma, ethics, and privacy; and finally, the systems’ applicability to real-world contexts. Specifically, we aim to (1) investigate whether these systems are designed using human-centered research methodologies and principles, as well as (2) generate concrete design guidelines to support the development of effective solutions in this context for future research. The following research questions will be addressed in our systematic review:

- Which measures of usability, engagement, and feasibility are currently used in substance use–centered mHealth studies? How is efficacy explicated in these studies?
- What key findings are reported in this literature, and to what extent do they apply to real-world contexts? Are the current approaches and systems scalable?
- Do these existing systems use human-centered design principles, and how is the presence or absence of human-computer interaction–based research methodologies associated with measures of usability and scalability?
- What are the common, persistent challenges faced by researchers and practitioners in developing mHealth systems for substance use, and how might they be addressed through robust, human-centered research techniques?
- How can we use findings, methods, and techniques from other areas of mHealth to inform future substance use detection and intervention work?

## Methods

### Study Design

To structure the design of this systematic review, we will use the PRISMA-P (Preferred Reporting Items for Systematic Review and Meta-Analysis Protocols) guidelines [11]. This methodology consists of literature search, article selection and screening, data extraction and analysis, and an assessment of study quality and bias.

### Search Strategy

We surveyed 4 large databases of digital health literature—ACM Digital Library, IEEE Xplore, JMIR, and PubMed—over a 5-year period (2016-2021), using keywords and terms extracted from an initial literature review. Search terms focused on 3 key areas: substance-related terms such as alcohol, cannabis, opioids, and tobacco; mHealth-related terms such as smartphone, smartwatch, and conversational agents; and design- and usability-related terms such as acceptability, user perspectives, engagement, and adoption. The full list of terms is provided in Table 1 and encompasses a broad survey of the existing literature on mHealth approaches for substance use that include some form of discussion on usability. Further, “similar articles” and citation networks were used to identify more relevant papers. This initial search yielded 3352 papers.

**Table 1:** Search terms for literature review.

Domain	Keywords
Substances of interest (searched for in the abstract or title of the articles)	Alcohol OR opioid OR “substance use” OR tobacco OR cannabis OR marijuana OR “substance abuse” OR “drug use” OR cigarette OR vaping OR smoking
Relevant mHealth^a^ platforms (searched for in all given metadata for an article)	mHealth OR mobile OR smartphone OR “mobile application” OR “smartphone application” OR wearable* OR smartwatch OR “conversational agent” OR “virtual coach” OR *bot OR smart-speaker OR “smart speaker”
Design techniques, evaluation methods, and user experience (searched for in the full text of the articles)	Usability OR “user centered” OR acceptability OR engagement OR “treatment adherence” OR adherence OR “user experience” OR acceptance OR “user acceptability” OR efficacy OR effectiveness OR “human-centered” OR “human centered” OR “user perspectives” OR “user perceptions” OR adoption OR feasibility

^a^mHealth: mobile health.

Substances of interest were searched for in the abstract or title of the articles, to maintain relevance with the aim of this work. Relevant mHealth platforms were searched for in all given metadata for an article, whereas terms related to design techniques, evaluation methods, and user experience were searched for in the full text of the article.

### Inclusion and Exclusion Criteria

Given that mHealth technologies in this domain span an extensive set of target populations, methodologies, and devices, we established appropriate inclusion and exclusion criteria to define the scope of this protocol.

We included papers that meet all 4 following criteria:

- Papers describing an mHealth system or app with a reasonable degree of implementation and deployment, including generating results from a user study of any scale. We specifically included apps and systems that were implemented and tested out by users, as one of our main research questions was to analyze user perspectives—and their influence on the system design—through all stages of development.
- Papers presenting an app deployed on ubiquitous devices like smartphones, smartwatches, wearables, or smart speakers.
- Papers including a discussion of any depth about user perspectives, design approaches, usability, acceptability, feasibility, engagement, ethics, or privacy and stigma concerns, as this was an important variable in our literature analysis.
- Papers or articles in the English language.

We excluded papers that matched the following criteria:

- Papers describing machine learning approaches without system deployment and user testing, as this would not align with our primary aims of assessing mHealth approaches.
- Papers presenting social media–based detection and intervention approaches, since they do not include systems that are device-specific.
- Works targeting associated mental illnesses and treating substance use as a symptom, consequence, or a distal measure or outcome, as we wanted to only focus on papers that included or assessed substance use proximally.
- Papers presenting analyses without system implementation and deployment (eg, works examining associations between substance use and crime, economy, etc, without an app description).
- Telehealth or telemedicine (ie, telephone and video technologies) and web-based approaches (eg, patient portals), as we wanted to focus only on mHealth approaches, that is, systems that based their detection or intervention mechanisms on data collected through mobile devices such as smartphones or wearables (eg, self-report, biological samples, location data, sensor data, or physiologic data).

### Data Extraction

All title- and abstract-screened articles were exported to a Zotero (version 5.0.96.3; Corporation for Digital Scholarship) library, and duplicate studies were removed. To extract relevant data from the selected list of papers, we will use standard Microsoft Excel forms that include the following variables: population targeted (eg, age group and existing health conditions), study type (eg, detection, data collection, feasibility or usability of the system or approach, intervention study, and app evaluation), study characteristics (eg, qualitative or quantitative methodology, number of participants, participant split, duration, and outcome domain), form of mHealth approach (eg, mobile phone, wearable, conversational agents, and combination), system description, targeted substance (eg, alcohol use, cannabis use, opioid use, tobacco use, and substance co-use), theoretical constructs used (eg, peer-based care facilitation or intervention, gamification, cognitive training, and behavioral theory), design approaches or the types of research that inform design (eg, focus groups, think-alouds, interviews, participatory design, observational studies, case studies, diaries, user testing, scenarios, or personas), rationale for design approach (eg, target population–based, behavior-based, or substance-based), design evaluation methods or how the efficacy of design was assessed (eg, through completion of tasks, usability ratings, interviews, usage duration data, or compliance data), design evaluation findings, privacy concerns and stigma-related findings, study outcomes or health and clinical outcomes (eg, frequency or intensity of substance use, mental health status, change in experiences or behaviors associated with substance use such as cravings, use of other substances, access to care, and change in consequences of substance use), and the cost of intervention.

### Study Quality Assessment

To assess the quality of the studies included in the final analyses, we will use the mHealth Evidence Reporting and Assessment [12] framework, which includes checklists consisting of numerous criteria deemed essential for reporting interventions and study design, such as intervention content, methods of delivery, usability testing, and user feedback, as well as other items such as discussions of population-level infrastructure availability and scalability limitations.

### Data Analysis

A descriptive analysis using the aforementioned data extraction forms will be conducted after a final list of papers has been selected. We will organize themes identified in our analysis through subgroups to provide a succinct discussion of existing literature. In this discussion, we will aim to elaborate upon the following: how effectiveness of approach was explicated in various studies; what outcomes were deemed most important for the success of the approach; the various design approaches and evaluation techniques that were used and the subsequent findings; scope of the studies analyzed; how findings from these studies can inspire future work in substance use, as well as in mHealth; and finally, the features of successful mHealth approaches used in other domains that can positively impact the scalability and usability of current systems and apps in problematic substance use detection, prevention, and treatment.

## Results

As of December 2021, we have identified 257 papers that met our initial screening. These papers will be further analyzed to remove those that do not exactly match the inclusion and exclusion criteria established above.

## Discussion

In this protocol, we detailed the plan for a systematic analysis of design and evaluation approaches in mHealth systems with the aim to address, prevent, and treat problematic substance use. We anticipate that studies that invest in rigorous and iterative design or evaluation methods, incorporate theoretical framing, and consider issues such as accessibility, scalability, privacy concerns, and social stigma will result in systems that elicit positive health outcomes.

In this review, our principal findings will be organized into the following 3 main parts:

- First, we will present an analysis of the types of human-centered design and evaluation approaches used in the current state-of-the-art mHealth work in substance use. This analysis will allow us to highlight the most frequently used methods in this domain; the insights these approaches are capable of generating; and the challenges they pose with respect to feasibility, reliability, and generalizability.
- Second, we will establish the main constructs on which usability evaluations have been based in the selected studies and highlight those that have so far received less attention in this domain. Hence, we aim to explore whether each study conducts evaluations based on measures of privacy, scalability, and sustained usability, and whether ethical implications are considered in the proposed systems. This analysis of constructs will illustrate the opportunities and gaps in the use of human-centered techniques to improve health outcomes in this domain.
- Third, we examine the various components of the systems themselves: the theoretical foundations that guide them, the passive and self-reported data collected, detection and intervention methods, intervention content, design elements used in the interface (eg, notifications, gestures, and animations), and the platforms on which they are deployed. Extracting and analyzing these aspects will emphasize the characteristics that contribute to the overall user experience.

Through these multifaceted findings, our eventual goal is to establish best practices and guidelines for human-centered mHealth systems that target substance use detection and interventions. These guidelines will span several aspects across the system design flow, including suitable theoretical frameworks that address substance use in the population of interest, design elements and practices that can effectively be used to sustain user engagement and provide a rich user experience, approaches to responsibly collect data and ensure users’ trust in the system, incorporation of evidence-based practices that improve health outcomes, and practices to ensure the system is accessible and operates on ethical principles. Future research into mHealth tools for substance use and subsequent implementations of apps can use these guidelines to develop systems that encourage meaningful use and support enduring impact.

Thus, this review will build on prior work in understanding the effectiveness of mHealth systems in this domain by broadening the scope from usability assessments to a more comprehensive understanding of user experience. Further, our work will also add to current literature in the human-computer intervention field by assessing the relative strengths of various design and evaluation approaches for mHealth systems.

There has been an increasing focus on developing novel mHealth systems to understand and address substance use due to their potential impact. This review aims to provide a comprehensive look at whether and how human-centered approaches are being used to create systems to address substance use; however, we are limited by the volume of new work constantly emerging in this domain. Thus, this review should not be treated as exhaustive but rather as a useful reference that can be used while considering various design and evaluation approaches in mHealth for a substance use context. Our work also has limitations due to its focus on ubiquitous devices, which means that other approaches such as social media and telehealth are not analyzed in this review. Lastly, we focus on works that assess substance use (and associated behaviors) as a primary outcome and exclude studies that assess substance use adjacent to other health issues, thus limiting the applicability of findings and generated guidelines to systems that solely target substance use. Future research could expand the scope of this work by including other modalities of mHealth that target a wide variety of physical and mental health issues related to substance use.

## Abbreviations

mHealth: mobile health
PRISMA-P: Preferred Reporting Items for Systematic Review and Meta-Analysis Protocols
